# Yi-Qi-Jian-Pi Formula Suppresses RIPK1/RIPK3-Complex-Dependent Necroptosis of Hepatocytes Through ROS Signaling and Attenuates Liver Injury *in Vivo* and *in Vitro*

**DOI:** 10.3389/fphar.2021.658811

**Published:** 2021-04-23

**Authors:** Feixia Wang, Li Tang, Baoyu Liang, Chun Jin, Liyuan Gao, Yujia Li, Zhanghao Li, Jiangjuan Shao, Zili Zhang, Shanzhong Tan, Feng Zhang, Shizhong Zheng

**Affiliations:** ^1^ Department of Integrated TCM and Western Medicine, Nanjing Hospital Affiliated to Nanjing University of Chinese Medicine, Nanjing, China; ^2^ Jiangsu Key Laboratory for Pharmacology and Safety Evaluation of Chinese Materia Medica, School of Pharmacy, Nanjing University of Chinese Medicine, Nanjing, China

**Keywords:** acute-on-chronic liver failure, reactive oxygen species, inflammation, hepatocytes necroptosis, Yi-Qi-Jian-Pi formula

## Abstract

Acute-on-chronic liver failure (ACLF) is described as a characteristic of acute jaundice and coagulation dysfunction. Effective treatments for ACLF are unavailable and hence are urgently required. We aimed to define the effect of Yi-Qi-Jian-Pi Formula (YQJPF) on liver injury and further examine the molecular mechanisms. In this study, we established CCl_4_-, LPS-, and d-galactosamine (D-Gal)-induced ACLF rat models *in vivo* and LPS- and D-Gal-induced hepatocyte injury models *in vitro*. We found that YQJPF significantly ameliorates liver injury *in vivo* and *in vitro* that is associated with the regulation of hepatocyte necroptosis. Specifically, YQJPF decreased expression of receptor-interacting protein kinase 1 (RIPK1), receptor-interacting protein kinase 3 (RIPK3) and pseudokinase mixed lineage kinase domain-like (MLKL) to inhibit the migration of RIPK1 and RIPK3 into necrosome. YQJPF also reduces the expression of inflammatory cytokines IL-6, IL-8, IL-1β, and TNF-α, which were regulated by RIPK3 mediates cell death. RIPK1 depletion was found to enhance the protective effect of YQJPF. Furthermore, we showed that YQJPF significantly downregulates the mitochondrial reactive oxygen species (ROS) production and mitochondrial depolarization, with ROS scavenger, 4-hydroxy-TEMPO treatment recovering impaired RIPK1-mediated necroptosis and reducing the expression of IL-6, IL-8, IL-1β, and TNF-α. In summary, our study revealed the molecular mechanism of protective effect of YQJPF on hepatocyte necroptosis, targeting RIPK1/RIPK3-complex-dependent necroptosis *via* ROS signaling. Overall, our results provided a novel perspective to indicate the positive role of YQJPF in ACLF.

## Introduction

Acute-on-chronic liver failure (ACLF) is a syndrome characterized by acute decompensation of chronic liver disease, which has a high short-term mortality rate, and it seriously threatens the lives of patients with chronic liver disease (Xiang et al., 2020). However, the pathogenesis of ACLF remains poorly understood. Among Asian countries, the prevalence of hepatitis in China is still high. There are approximately 97 million hepatitis B virus (HBV) carriers and one million hepatitis C virus (HCV)-infected patients in China. Chronic viral hepatitis is still the basis in ACLF (Xie et al., 2019). Virus replication and bacterial infection are the main causes of liver failure. However, no effective therapy for liver failure beyond supportive treatment is currently available; hence, a new treatment or medicine against liver failure is needed. Previous studies have suggested that ACLF develops in patients with cirrhosis as a consequence of precipitating events leading to acute decompensation and multi-organ failure (Sarin and Choudhury, 2016). Despite variations in the definition of ACLF in different regions, progressive, unrelenting hepatocyte injury and death are common hallmarks of ACLF, which have been well-documented as pathobiology of ACLF. Effective treatments to target hepatocyte injury and death due to ACLF are lacking and thus are urgently required. Unlike apoptosis and pyroptosis, necroptosis is a caspase-independent death program (Asrani et al., 2019). The dying cells exhibit none of the morphological characteristics of apoptosis, instead these cells display the swelling associated with necrosis; the dying cell can trigger an innate immune response, cause the release of inflammatory cytokines, and exacerbate tissue damage (Wang et al., 2014). The inhibitors of necroptosis could have therapeutic potential.

Traditional Chinese medicines have received attention worldwide in last few decades because of their satisfactory therapeutic effects, facile availability, and cost-effectiveness (Bajaj et al., 2018; Engelmann et al., 2020). Our previous study confirmed that the treatment with Yi-Qi-Jian-Pi Formula (YQJPF) composed of Huangqi, Taizishen, Baizhu, Chenpi, Danggui, Fulin, Huangqin, and Gancao can ameliorate HBV-ACLF. *Corethrodendron multijugum* (Maxim.) B. H. Choi and H. Ohashi (Huangqi in Chinese) and Pseudostellariae Radix, *Pseudostellaria heterophylla* (Miq.) Pax (Taizishen in Chinese) are Monarch drugs; Angelicae Sinensis Radix, *Angelica sinensis* (Oliv.) Diels (Danggui in Chinese), *Wolfiporia extensa* (Peck) Ginns (Fulin in Chinese), *Atractylodes lancea* (Thunb.) DC (Cangzhu in Chinese) and *Atractylodes macrocephala* Koidz (Baizhu in Chinese) are Ministerial drugs; and Citrus Reticulata, Citrus × aurantium L. (Chenpi in Chinese), Scutellariae Radix *Scutellaria baicalensis* Georgi (Huangqin in Chinese), and licorice, *Glycyrrhiza uralensis* Fisch. ex DC., G. glabra L., and G. inflata Batalin (Gancao in Chinese) are adjuvants. A retrospective cohort study on 60 patients with HBV-ACLF reported that YQJPF could prevent the development of liver failure and improve the Model for End-Stage Liver disease score (Zhang Z. et al., 2014; Lian et al., 2015). However, the regulation mechanism of its effect has not been elucidated. Atractylone is the main sesquiterpenic constituent of *Atractylode japonica*, which is a necessary component of YQJPF. Atractylode japonica was used to treatment of several diseases such as rheumatic diseases, digestive disorders, hepatic protection and influenza for a long history in China. Additionally, atractylone was reported to have anti-inflammatory and anti-hepatotoxic effects (Cheng et al., 2019).

In the present study, we developed *in vivo* and *in vitro* models with d-galactosamine (D-gal)/LPS injection that recapitulate some features of clinical ACLF. In these models, we assessed the features of ACLF in laboratory settings. Further, ACLF models were treated with YQJPF to assess its effect on liver injury. Additionally, we explored the role of inflammation infiltration and hepatocyte necroptosis and the potential mechanisms for the treatment of liver injury in ACLF.

## Materials and Methods

### Reagents and Antibodies

Methylprednisolone was obtained from the Nanjing Hospital affiliated to Nanjing University of Chinese Medicine (Nanjing, Jiangsu China) and was dissolved in PBS to a concentration of 30 mg/ml and was stored at 4°C. Necrotatin-1 (Nec-1) and 4-hydroxy-TEMPO (Tempol) were bought from Sigma-Aldrich (St Louis, MO, United States). Dulbecco’s modified essential medium (DMEM), fetal bovine serum (FBS), Opti-MEM medium, phosphate-buffered saline (PBS), and trypsin-EDTA were purchased from GIBCO (Grand Island, NY, United States). The antibodies against *β*-actin (20536-1-AP), RIPK1 (17519-1-AP), and RIPK3 (17563-1-AP) were purchased from Proteintech Group, Inc (Rosemont, IL, United States). Caspase-3 (#9662), cleaved-caspase-3 (#9664), caspase-8 (#9746), cleaved-caspase-8 (#9748), TNF-α (#11948), IL-18 (#54943), IL-1β (#12242), and IL-6 (#12153) were procured from Cell Signaling Technology (Danvers, MA, United States). Mixed lineage kinase domain-like protein (MLKL; ab243142) were purchased from Abcam Technology (Abcam, Cambridge, United Kingdom).

### Preparation and High-Performance Liquid Chromatography of YQJPF for Quality Control

YQJPF was provided by Nanjing University of Chinese Medicine, Nanjing Hospital Affiliated to Nanjing University of Chinese Medicine. YQJPF was prepared from nine commonly used Chinese herbal medicines (Table 1). All the herbal constituents were obtained from the Beijing Tong Ren Tang Co. Ltd. (Beijing, China). Huangqi, Taizishen, Baizhu, Chenpi, Danggui, Fulin, Huangqin, and Gancao were mixed in the ratio 30:30:30:10:15:15:3:10:10.

**TABLE 1:** The composition of traditional Chinese medicine in Yi-Qi-Jian-Pi Formula (YQJPF).

Components	Latin name	Medical parts	Amount used (g)	Percentage (%)
Huangqi	*Corethrodendron multijugum* (Maxim.) B. H. Choi and H. Ohashi	Root	30	19.6
Taizishen	*Pseudostellaria heterophylla* (Miq.) Pax	Root	30	19.6
Danggui	*Angelica sinensis* (Oliv.) Diels	Rhizome	30	19.6
Baizhu	*Atractylodes macrocephala* Koidz	Root	15	9.8
Fulin	*Wolfiporia extensa* (Peck) Ginns	Sclerotium	15	9.8
Cangzhu	*Atractylodes lancea* (Thunb.)DC	Fruit	10	6.5
Chenpi	*Citrus* × aurantium L.	Pericarp	10	6.5
Huangqin	*Scutellaria baicalensis* Georgi	Root	10	6.5
Gancao	*Glycyrrhiza uralensis* Fisch. ex DC., G. glabra L., and G. inflata Batalin	Root, hizome	3	2.1

**FIGURE 1:**
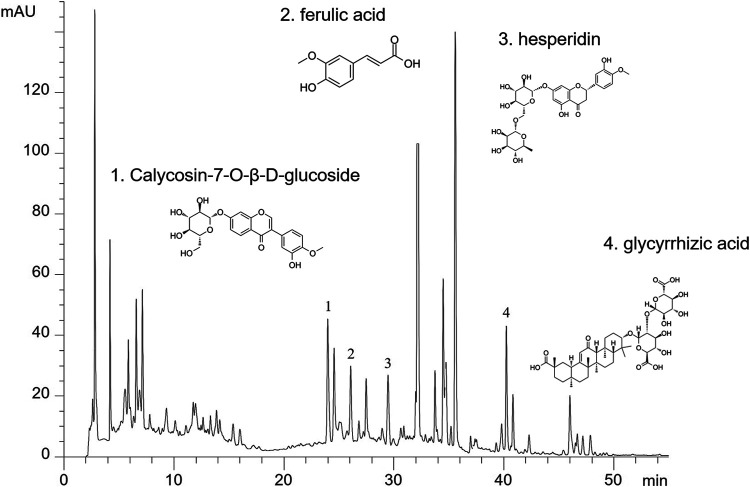
Establishment of a new model of ACLF in rats and the experimental procedures. Rats were randomly divided into the following seven groups (eight rats in each group).

Then, all herbs were decocted twice and the decoction liquids were concentrated to a density of 2.86 g/ml and was stored at 4°C. For quality control, HPLC analysis was used as previously described (Yao et al., 2018). YQJPF were assessed using an Agilent 1,260 liquid chromatography system (United Kingdom). Briefly, 10 μl YQJPF solution was injected into an apparatus with an auto sampler. Chromatographic separation was implemented at a flow rate of 1 ml/min with an Agilent C18 column (4.6 mm × 250 mm, 5 μm). The separation phase was composed of 0.2% phosphoric acid (A) and acetonitrile (B). The linear concentration solution gradually increases from 2 to 72% of solvent B over the course of 45 min. The separation temperature was 40°C, with a detection wavelength of 254 nm. The results are shown in Figure 1.

### Animals and Experimental Design

**FIGURE 2:**
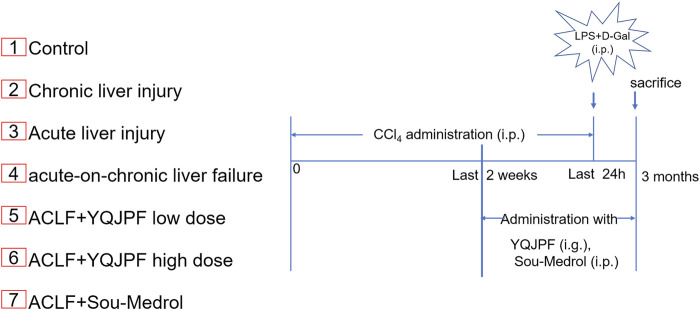
The major components in YQJPF were assessed using HPLC. Calycosin-7-O-β-D-glucoside, ferulic acid, hesperidin, and glycyrrhizic acid were found to be in the amounts of 0.3676, 0.6639, 2.442, and 0.4028 mg/ml, respectively.

A total of 56 male Sprague-Dawley rats, with the body weight of 180–220 g, were purchased from Beijing Vital River Laboratory Animal Technology Co. Ltd (Beijing, China). All animals were housed with laboratory chow and tap water at 22 ± 2°C under 55 ± 5% humidity-controlled environment and a 12-h light–dark cycle. The experimental design was approved by the Institutional and Local Committee on the Care and Use of Animals of Nanjing University of Chinese Medicine (Nanjing, China). All animals were provided care in compliance with the National Institutes of Health (United States) guidelines. A mixture of CCl_4_ and olive oil [1:1 (w/v)] was used to induce liver cirrhosis in rats (1.5 ml/kg body weight). LPS (100 μg/kg) and D-Gal (400 mg/kg) were used to induce acute liver injury. The mice were randomly divided into seven groups (*n* = 8 for each group). The experimental groups were as follows (Figure 2):

Group 1: control group; i. p. with olive oil.
Group 2: chronic liver injury model group; i. p. with CCl_4_ for 12 weeks.
Group 3: acute liver injury model group; i. p. with LPS and D-Gal once.
Group 4: ACLF model group; i. p. with CCl_4_ for 12 weeks followed by i. p. with LPS and D-Gal once.
Group 5: YQJPF low-dose treatment group; i. p. with CCl_4_ for 12 weeks and i. g. with YQJPF (14.3 g/kg) for 2 weeks followed by i. p. with LPS and D-Gal once.
Group 6: YQJPF high-dose treatment group; i. p. with CCl_4_ for 12 weeks and i. g. with YQJPF (28.6 g/kg) for 2 weeks followed by i. p. with LPS and D-Gal once.
Group 7: methylprednisolone treatment group; i. p. with CCl_4_ for 12 weeks and i. p. with methylprednisolone (15 mg/kg) for 2 weeks followed by i. p. with LPS and D-Gal once.

### Cell Culture

Human LO2 cell line was obtained from the Cell Bank of Chinese Academy of Sciences (Shanghai, China). Cells were cultured in DMEM with 10% FBS and 1% antibiotics and were incubated in 5% CO_2_ and 95% air humidified atmosphere at 37°C.

### Liver Histopathology

Liver hematoxylin and eosin (H&E) and Masson and Sirius Red staining were performed as per the previously described methods (Jin et al., 2016). Representative pictures of liver sections were displayed.

### Serum Biochemistry

Blood samples were collected and incubated at room temperature for 1 h, and extracted serum was collected after centrifugation. Levels of alkaline phosphatase (ALP), aspartate aminotransferase (AST), and alanine aminotransferase (ALT) in serum were measured using enzyme-linked immunosorbent assay kits (Nanjing Jinting Bioengineering Institute, Nanjing, China) according to the manufacturer’s protocols.

### Determination of Hepatic mtDNA, ATP, Malondialdehyde, and Superoxide Dismutase Levels

Liver tissues were treated according to the treatment regimen mentioned earlier After treatment, hepatic mtDNA, ATP, malondialdehyde (MDA), and superoxide dismutase (SOD) were quantified using corresponding kits (Nanjing Jinting Bioengineering Institute, Nanjing, China) according to the manufacturer’s instructions.

### Immunohistochemistry

Immunohistochemical staining of liver tissue was performed using antibodies against caspase 8, MLKL, RIPK1, and RIPK3, as previously described (Zhang F. et al., 2014). Representative pictures of liver sections were displayed.

### Cell Viability Assay

To assess the effects of different treatments on cell proliferation, Cell viability analysis was performed using MTT. In brief, The LO2 cells were seeded into 96-well plates at a density of 3 × 10^3^ cells per well. After incubating for 24 h, the cells were treated with LPS (10 μg/ml) and D-Gal (10 mg/ml) for 4 h for the hepatocyte injury model. Further, the cells were treated with serial concentration gradients of YQJPF and atractylone for another 24 h, with triplicates for each concentration. Then 5 mg/ml MTT was added in cells, and cells were incubated at 37°C for 4 h, and the absorbance was measured at a wavelength of 490 nm. Independent experiments were performed in triplicates.

### Trypan Blue Staining

Cell death was determined using the trypan blue staining assay (Beyotime Biotechnology, Shanghai, China). In brief, after YQJPF treatment, the cells were digested using trypsin without EDTA followed by centrifugation. The cells were further washed twice with PBS and were resuspended in PBS. Cell suspension and trypan blue solution were mixed gently in 1:1 ratio and allowed to stand for 3 min. A few of the stained cells were counted with a hemocytometer, and photographs were taken in a blinded fashion at random fields. Stained blue cells represented dead cells.

### RNA Isolation and Real-Time PCR

Total RNA was extracted from liver tissues and LO2 cells by using Trizol reagent according to the manufacturer’s protocol (Sigma-Aldrich, St. Louis, MO, United States) and further reverse-transcribed into cDNA by using PrimeScript RT reagent kit (TaKaRa Biotechnology, Beijing, China). Real-time PCR was performed using the SYBR Green I fluorescent dye (TaKaRa Biotechnology, Beijing, China), according to the manufacturer’s protocol. Glyceraldehyde phosphate dehydrogenase (GAPDH) served as an invariant control, and mRNA levels were expressed as fold changes after normalizing to GAPDH. The experiment was performed in triplicates. Primers (Genscript, Nanjing, China) are listed in Table 2 below.

**TABLE 2:** Primers used for human LO2 cells.

Gene	Forward sequence	Reverse sequence
IL-6	GCT​TCC​CTC​AGG​ATG​CTT​GT	ATT​AAC​TGG​GGT​GCC​TGC​TC
TNF-α	GAG​ACA​GAT​GTG​GGG​GGT​GTG​AG	TCC​TAG​CCC​TCC​AAG​TTC​CA
IL-1β	AGC​CAT​GGC​AGA​AGT​ACC​TG	TGA​AGC​CCT​TGC​TGT​AGT​GG
IL-18	CGA​GGG​AGT​GAA​GAC​CCT​G	TGG​GCG​TAA​GCT​TGG​AAT​GT
GAPDH	TGT​CAT​GGC​AGA​AGT​ACC​TG	GTT​AAC​TGG​GGA​GCC​TGC​TC

### Western Blot

Cells and liver samples were lyzed using a mammalian lysis buffer (Sigma St. Louis, MO, United States) and were denatured in SDS loading buffer. Then, western blot analysis was performed according to the manufacturer’s instructions (Bio-Rad, Hercules, CA, United States). Thereafter, Cells and liver samples were incubated overnight with antibodies against caspase 3, cleaved-caspase-3, caspase 8, cleaved-caspase-8, MLKL, RIPK1, RIPK3, IL-6, IL-18, IL-1β, and TNFα at 4°C. Protein detection, band visualization, and quantification were performed as per the manufacturer’s instructions. Anti-β-actin antibody (Danvers, MA, United States) was used as a loading control.

### Immunoprecipitation Assay

The immunoprecipitation assay was performed using co-immunoprecipitation (Co-IP) kit (Thermo) as per manufacturer’s instructions. Briefly, the cell lysates were incubated with anti-RIPK1 and anti-RIPK3 antibodies. Protein G-agarose beads were added and incubated overnight to precipitate the protein complexes. Western blot analysis was performed to detect the protein complexes expression.

### Immunofluorescence Analysis

LO2 cells were seeded on 24-well plates and cultured in DMEM with 10% FBS. Afterward, they were treated with corresponding reagents for 24 h. Then, the cells were fixed with 4% PFA for 30 min at 37°C, permeabilized with PBS-T (0.1% Triton x-100 dissolved in PBS), and blocked with PBS-B (4% BSA dissolved in PBS). The cells were stained with the corresponding antibody (1:200 dilution) overnight at 4°C, followed by incubation with FITC-labelled goat anti-rabbit IgG (1:100 dilution) for 2 h. Finally, 4′,6-diamidino-2-phenylindole (DAPI) staining was performed by incubation in dark for 5 min, and the fluorescence was observed using a fluorescence microscope (Nikon, Tokyo, Japan) to visualize the nuclei.

### Enzyme-Linked Immunosorbent Assay (ELISA)

Levels of IL-6, IL-8, IL-1β, and TNF-α in serum were measured using ELISA kits (Nanjing Jiancheng Bioengineering Institute, Nanjing, China) according to the manufacturer’s instructions.

### Intracellular ROS Assay

The level of intracellular ROS was determined using an oxidation-sensitive fluorescent probe, 2′,7′-dichlorodihydrofluorescin diacetate (DCFH-DA) (Beyotime Biotechnology, Shanghai, China). Briefly, LO2 cells were seeded in a 24-well plate and pre-treated with ROS scavenger Tempol (10 μM) for 1 h before exposing to YQJPF or selective RIPK1 inhibitor Nec-1. DCFH-DA probe was subsequently incubated with LO2 for 30 min. Subsequently, ROS levels were detected according to the manufacturer’s instructions.

### Determination of Mitochondrial ROS

We determined mitochondrial ROS production by using a fluorescent probe, MitoSOX, which is a red mitochondrial superoxide indicator for live cell imaging (Molecular Probes; Life technologies). Briefly, LO2 cells were treated with YQJPF or selective RIPK1 inhibitor Nec-1. Further, LO2 cells were incubated with MitoSox reagent (5 μM) for 10 min at 37°C and washed with PBS. The red fluorescence density was detected using a fluorescence microscope (Nikon, Tokyo, Japan) with the rhodamine channel.

### Mitochondrial Membrane Potential Assay

The mitochondrial membrane potential (MMP) was determined using 5.5′,6.6′-tetrachloro-1.1′,3.3′-tetraethylbenzimidazolylcarbocyanine iodide (JC-1) (Beyotime Biotechnology, Shanghai, China) staining as per the manufacturer’s protocol. LO2 cells were washed with PBS and incubated with JC-1 working solution at 37°C in dark for 20 min. After removing JC-1 solution, the cells were washed with PBS, and images were taken using a fluorescence microscope (Nikon, TiE, Japan) with both red and green channels. The MMP is represented by the average red/green fluorescence intensity ratio.

### Transmission Electron Microscopy

LO2 cells were seeded in six-well plates (14,000 cells/well). After the corresponding treatment, transmission electron microscopy (TEM) images were obtained using a JEM 1010 transmission electron microscope (JEOL, Tokyo, Japan). Briefly, LO2 cells were fixed in Karnovsky fixative solution (2% paraformaldehyde and 2.5% glutaraldehyde in 0.15 mol/L sodium cacodylate buffer, pH 7.1–7.3) during 1 h and then washed three times in a cacodylate buffer for 15 min. Then pellets were embedded into 2% agar and postfixed in 1% osmium tetraoxide in a cacodylate buffer during 1 h. Samples were then dehydrated in 50, 70, 95, and 100% of acetone for 2, 10, 30, and 60 min, respectively, and embedded into Durcupan ACM (Fluka, Analytical Sigma-Aldrich; Switzerland). And the ultrathin sections (70 nm) were placed on nickel grids and examined at 100 keV. For each group, electromicrographs were recorded at a microscope magnification of ×10,000.

### Flow Cytometer Analysis

FITC Annexin V apoptosis detection kit (BD Pharmingen™, BD Biosciences) was used for detection of cell apoptosis. Briefly, LO2 cells were seeded in six-well plates in complete medium and allowed to attach for 24 h. Then, cells were treated as indicated. Cells were further incubated for 24 h, in 5% CO_2_ at 37°C. After that, cells were trypsinized, washed in PBS and stained according to kit according to the manufacturer’s instructions. Stained cells were analyzed using BD Accuri C6 flow cytometer (BD Pharmingen™, BD Biosciences) and data was processed with FlowJo_v10.6.2 software. Each sample was assessed using a collection of 10,000 events. The mean values and standard deviations were calculated from three independent experiments.

### Statistical Analysis

Two-tailed Student’s tests and one-way ANOVA analysis were performed using GraphPad Prism software v. 7.0 (Graph Pad Software Inc., San Diego, CA). The values and data are presented as the mean ± SEM from three independent experiments. A *p* value of <0.05 was considered statistically significant.

## Results

### YPJPF Alleviated Liver Injury *In Vivo*

**FIGURE 3:**
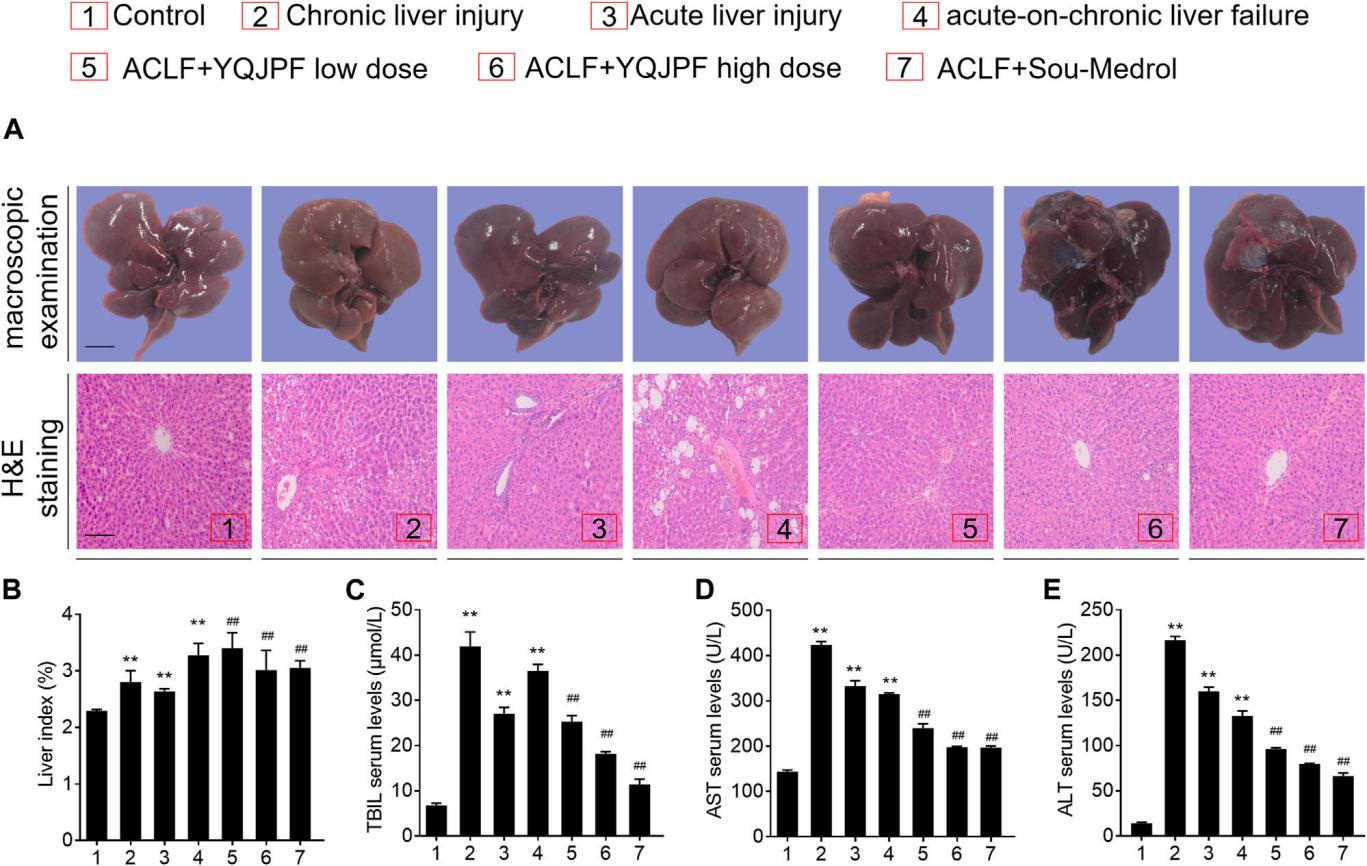
YPJPF alleviated liver injury *in vivo.* **(A)** Ratio of liver weight and body weight. **(B–D)** TBIL, AST, and ALT levels in serum. **(E)** Representative pictures of liver tissues and photograph of H&E-stained sections (Scale bars, 100 μm). Data are expressed as mean ± SD (*n* = 8) *vs. control group, *vs. ACLF group; *p < 0.05, **p < 0.01; ^#^ *p* < 0.05, ^##^ *p* < 0.01.

The ACLF rat model was pre-established through intraperitoneal injection of CCl_4_ for 12 weeks. Further, acute liver injury was induced by using a combination of LPS and D-Gal. And YQJPF was given for 2 weeks to investigate the protective effect on ACLF rats. In addition, methylprednisolone (Sou-Medrol) was used as a positive control drug, which has been confirmed as a therapeutic agent for ACLF (Zhao et al., 2012; Jia et al., 2020). And the establishment of a new model of ACLF in rats and the experimental procedures as shown in Figure 2. The livers in ACLF model became small and hard accompanied by necrosis, with small nodules on their surface; however, YQJPF and methylprednisolone were found to effectively ameliorate the liver morphology. Pathological examinations were used to manifest the effects of YQJPF on hepatic injury. As shown in H&E staining, YQJPF and methylprednisolone ameliorated the disordered hepatic structure (Figure 3A). The effect of YQJPF on liver injury was evaluated. Detection of serum biochemical indicators indicated improvement in liver injury indices such as TBil, ALT, and AST contents after 2 weeks of YQJPF and methylprednisolone treatment in rats with ACLF (Figures 3B–E). In summary, YQJPF and methylprednisolone significantly ameliorated liver injury in ACLF model rats.

### Protective Effect of YQJPF on LPS- and D-Gal-Induced Hepatocyte *In Vitro*

**FIGURE 4:**
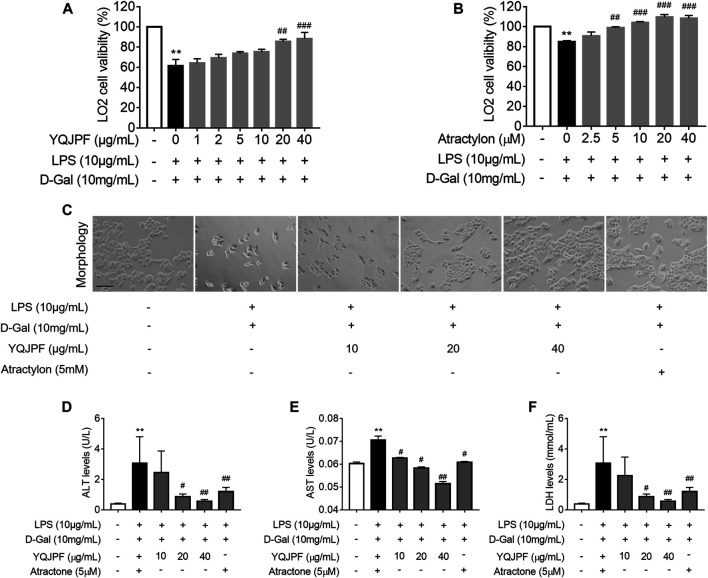
Protective effect of YQJPF on LPS- and D-Gal-treated hepatocytes *in vitro*. Human LO2 cells were treated with LPS + D-Gal for 4 h and further treated with YQJPF and atractylone at indicated concentrations. **(A,B)** MTT assay to study LO2 cell viability. **(C)** Cell morphology assessment. Scale bar, 200 μm. **(D–F)** ALT, AST, and LDH levels in LO2 cell culture. Data are expressed as mean ± SD (*n* = 6). *vs. control group, ^#^vs. LPS + D-Gal group; *p < 0.05, **p < 0.01; ^#^ *p* < 0.05, ^##^ *p* < 0.01.

Generally, massive hepatocyte death is implicated in ACLF. Here, we used LPS (10 μg/ml) and D-Gal (10 mg/ml) to induce liver injury in LO2 cells. MTT assay showed that LPS and D-Gal inhibit cell viability, whereas YQJPF and atractylone could promote cell viability in a dose-dependent manner. YQJPF above 10 μg/ml and atractylone at 5 μM showed a significant effect (Figures 4A,B). Light microscopy indicated that the adherent LO2 cells are swollen, and the cell morphology changed after LPS and D-Gal treatment for 4 h. YQJPF and atractylone could improve microscopic performance (Figure 4C). Levels of AST and ALT were detected to assess the hepatocytic damage. The levels of ALT, AST, and LDH in the supernatant of cultured LO2 cells were detected by ELISA. Results showed significantly increased levels of ALT, AST, and LDH in the model group and decreased levels of those in YQJPF and atractylone treatment groups (Figures 4D–F). Thus, YQJPF and atractylone exerted a protective effect on LPS- and D-Gal-induced hepatocytic injury.

### YQJPF Inhibited Necroptosis but Not Apoptosis on LPS- and D-Gal-Induced Hepatocytic Injury *In Vitro*

**FIGURE 5:**
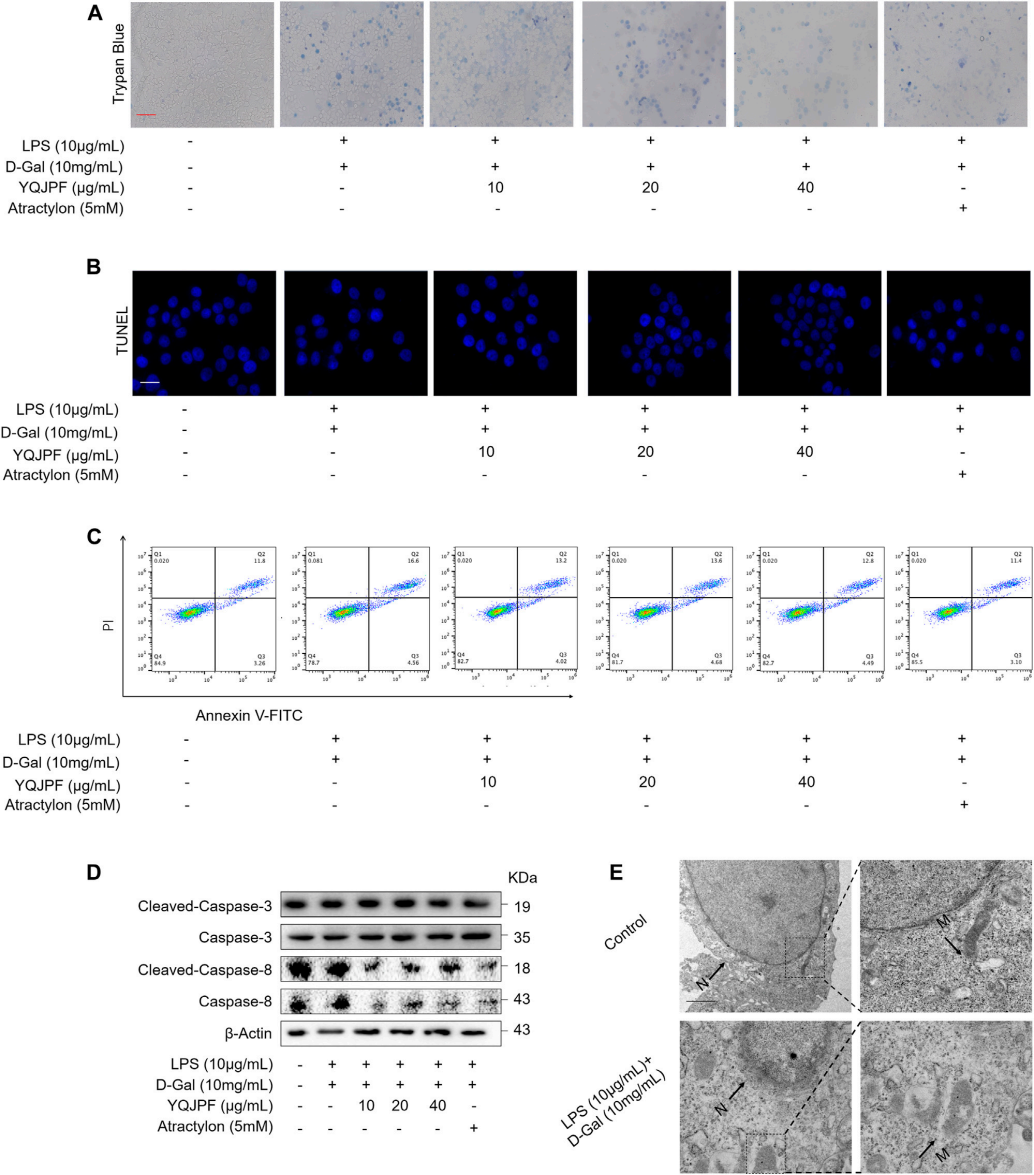
YQJPF inhibited hepatocytic cell death, which was not by apoptosis, in LPS- and D-Gal-treated LO2 cells *in vitro*. Human LO2 cells were treated with LPS + D-Gal for 4 h and further with YQJPF and atractylone. **(A)** Trypan blue staining to detect cell death. Scale bar, 100 μm. **(B)** TUUNEL staining to detect cell apoptosis. **(C)** Western blot analysis of the protein expression of caspases three and eight and cleaved caspases three and eight in the treated human LO2 cells. **(D)** cell apoptosis assay by flow cytometer in the treated human LO2 cells. **(E)** Transmission electron microscopy (TEM) of cells. Black arrows represent swollen mitochondria (M), whereas white arrows represent condensed and marginated chromatins in nuclei (N). Data are expressed as mean ± SD (*n* = 3). *vs. control group, ^#^vs. LPS + D-Gal group; *p < 0.05, **p < 0.01; ^#^ *p* < 0.05, ^##^ *p* < 0.01.

**FIGURE 6:**
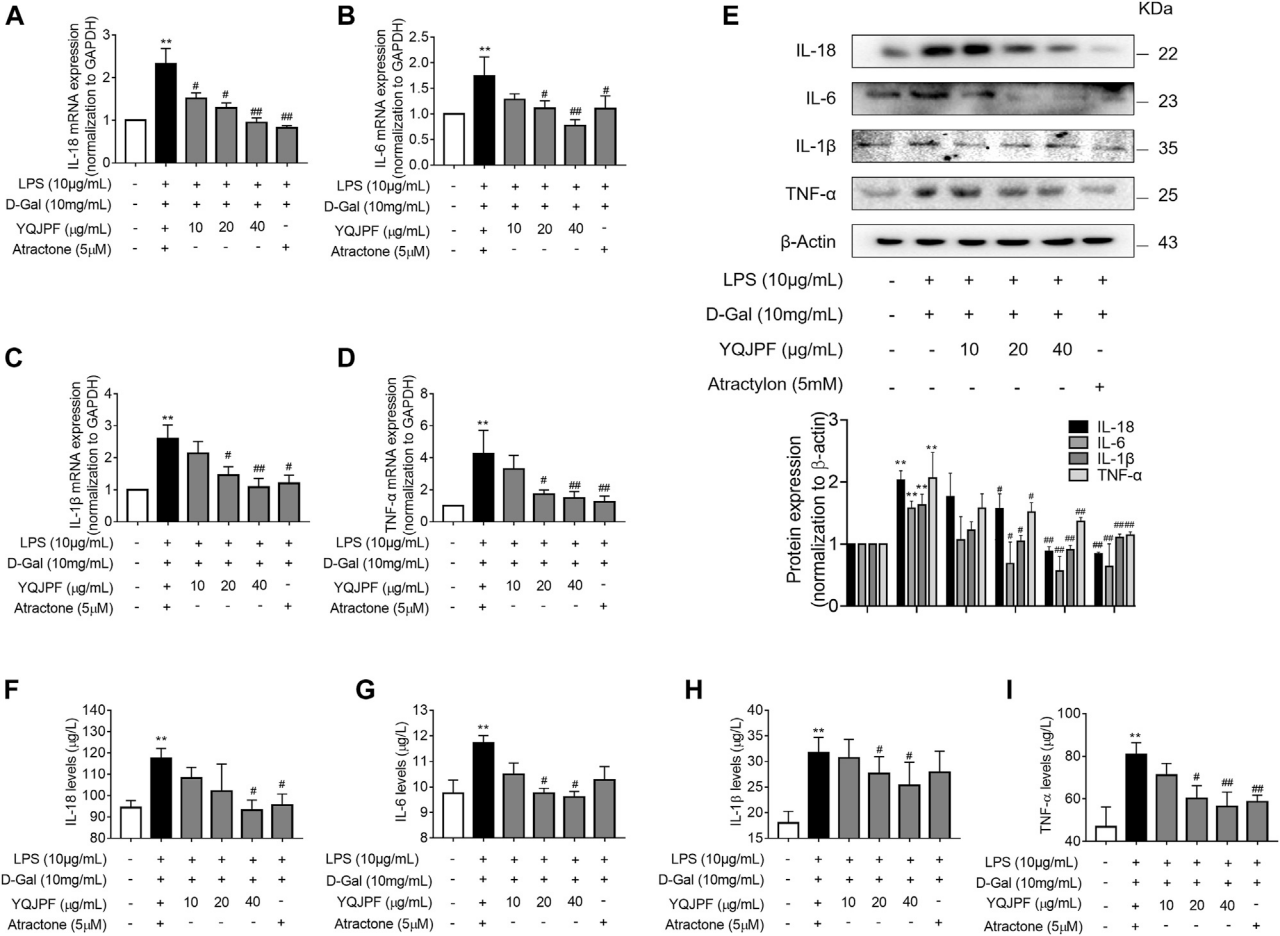
YQJPF inhibited necroptosis of LPS- and D-Gal-treated hepatocytes *in vitro*. Human LO2 cells were treated with LPS + D-Gal for 4 h and further with YQJPF and atractylone. **(A–D)** Real-time PCR analyses of genes of pro-inflammatory cytokines IL-1β, IL-6, TNF-α, and IL-18. **(E)** Western blot analysis and quantitative analysis of the expression of pro-inflammatory cytokines IL-1β, IL-6, TNF-α, and IL-18 (F–I) Expression levels of IL-1β, IL-6, TNF-α, and IL-18 in LO2 were detected by ELISA. Data are expressed as mean ± SD (*n* = 3). *vs. control group, ^#^vs. LPS + D-Gal group; *p < 0.05, **p < 0.01; ^#^ *p* < 0.05, ^##^ *p* < 0.01.

Necrosis and apoptosis are the most common pathways of death during liver injury (Schwabe and Luedde, 2018). Hence, we first examined which pathway plays a significant role in LPS- and D-Gal-induced hepatocytic injury. The result of the TUNEL assay indicated that YQJPF (10, 20, and 40 μg/ml) and atractylone (5 μM) have no effect on apoptosis (Figure 5B). The protein expression of key indicators such as caspase 3, caspase 8, and the cleaved caspase-3 and caspase-8 did not change after LPS and D-Gal treatment (Figure 5C). Then, the cell apoptosis was determined by flow cytometry, and results indicated that YQJPF and atractylone have no effect on apoptosis (Figure 5D). Therefore, LPS- and D-Gal-induced cell death was not dependent on apoptosis because no obvious apoptotic features could be observed. YQJPF and atractylone prevented cell death without influencing apoptosis. Next, we assessed whether necroptosis plays a role in this process. Positive result in trypan blue staining indicated that membrane permeability is altered by LPS and D-Gal. YQJPF or atractylone treatment significantly decreased the number of dead cells (Figure 5A). To confirm our findings, we used TEM analysis to observe cell morphology and structure. The cells exhibited a typical necrotic cell death morphology, including swelling of organelles (especially mitochondria), condensation of chromatin into small, irregular patches, and chromatin margination, after LPS and D-Gal treatment (Figure 5E). YQJPF could restore cell morphology and structure in a dose-dependent manner. Atractylone exhibited the same effect. Necroptotic cells underwent organelle breakdown, leading to the leakage of intracellular contents consequently triggering inflammation (Weinlich et al., 2017). Further, we examined some inflammation-associated biomarkers. mRNA and protein levels of pro-inflammatory cytokines IL-1β, IL-6, TNF-α, and IL-18 were detected using RT-qPCR, ELISA, and western blot analysis (Figures 6A–I). The results showed that pro-inflammatory cytokines are decreased in YQJPF and atractylone treatment groups compared with the LPS- and D-Gal-induced model group. Above all, we demonstrated that LPS and D-Gal induce hepatocyte necroptosis but not apoptosis and that YQJPF and its active ingredient atractylone could protect hepatocytes from necroptosis by reducing the expression of inflammatory cytokines in hepatocytes.

### The RIPK1/RIPK3 Complex Is Required for YQJPF to Inhibit Necroptosis *In Vitro*

**FIGURE 7:**
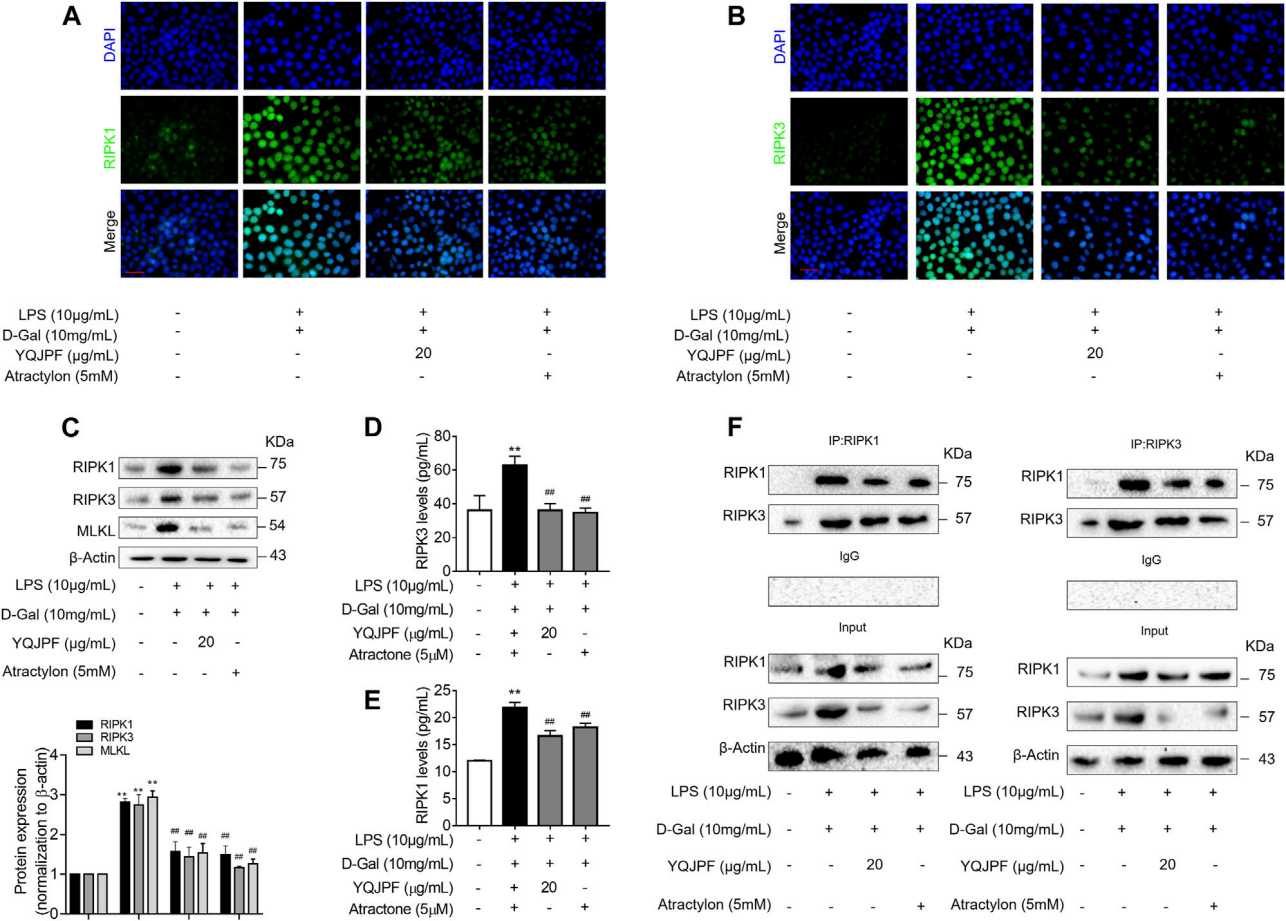
The RIPK1/RIPK3-complex is required for YQJPF to inhibit hepatocyte necroptosis *in vitro*. Human LO2 cells were treated with LPS + D-Gal for 4 h and further with YQJPF and atractylone. **(A,B)** Immunofluorescence staining of RIPK1 and RIPK3 in LO2 cells. Scale bar, 50 μm. **(C)** Western blot analysis and quantitative assessment of RIPK1, MLKL and RIPK3. **(D,E)** Expression levels of RIPK1 and RIPK3 in LO2 were detected by ELISA. **(F)** RIPK1 was immunoprecipitated with its antibody and resulted in co-immunoprecipitation of RIPK3. Immunoprecipitation of RIPK3 with its antibody caused co-immunoprecipitation of RIPK1 in LO2 cells. Data are expressed as mean ± SD (*n* = 3). *vs. control group, ^#^vs. LPS + D-Gal group; *p < 0.05, **p < 0.01; ^#^ *p* < 0.05, ^##^ *p* < 0.01.

**FIGURE 8:**
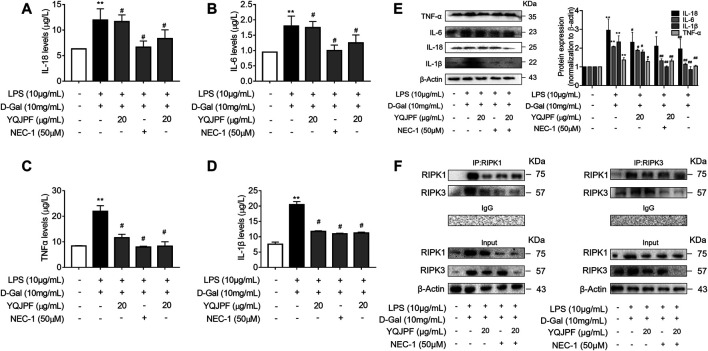
YQJPF inhibited hepatocyte necroptosis in RIPK1-dependent manner *in vitro*. Human LO2 cells were treated with LPS + D-Gal for 4 h and further pre-treated with 50 μM Nec-1 for 1 h, followed by YQJPF or atractylone treatment for 24 h. **(A–D)** Expression levels of IL-1β, IL-6, TNF-α, and IL-18 in LO2 cells were detected by ELISA. **(E)** Western blot and quantitative analysis of IL-1β, IL-6, TNF-α, and IL-18. **(F)** RIPK1 was immunoprecipitated with its antibody and resulted in co-immunoprecipitation of RIPK3. Immunoprecipitation of RIPK3 with its antibody caused co-immunoprecipitation of RIPK1 in LO2 cells. Data are expressed as mean ± SD (*n* = 3). *vs. control group, ^#^vs. LPS + D-Gal group; *p < 0.05, **p < 0.01; ^#^ *p* < 0.05, ^##^ *p* < 0.01.

The RIPK1/RIPK3 signaling pathway has been reported to be involved in the induction of necroptosis (Lin et al., 2016; Newton et al., 2016). Thus, we investigated using western blot and ELISA whether YQJPF and atractylone alter the expression of RIPK1, RIPK3 and MLKL. As shown in (Figures 7A–E), YQJPF or atractylone treatment downregulated the expression of RIPK1, RIPK3 and MLKL. Immunofluorescence staining of RIPK1 and RIPK3 further validated these results. The association between RIPK1 and RIPK3 was analyzed using immunoprecipitation, which indicated that YQJPF and atractylone could inhibit the interaction between RIPK1 and RIPK3 (Figure 7F). To identify whether YQJPF and atractylone are dependent on RIPK1/RIPK3-mediated necroptosis, we used the RIPK1 kinase inhibitor necrostatin-1 (Nec-1) to verify the effect of YQJPF and atractylone. The results indicated that Nec-1 and YQJPF work together, and they had the most significant inhibitory effect on LO2 necroptosis induced by LPS and D-Gal (Figures 8A–E). We further investigated the interaction between RIPK1 and RIPK3 in YQJPF-treated LO2 cells through immunoprecipitation. RIPK1 and RIPK3 were significantly immunoprecipitated in LPS- and D-Gal-induced cells, which could be impaired by YQJPF and RIPK1 inhibitor Nec-1 (Figure 8F). Overall, YQJPF could downregulate the expression of RIPK1 and RIPK3, inhibit their aggregation to form necrosome, and eventually reduce the expression of pro-inflammatory cytokines by inhibiting RIPK1/RIPK3-mediated necroptosis.

### YQJPF Inhibited Hepatocyte Necroptosis Through Inhibition of Mitochondrial ROS Generation and Depolarization *In Vitro*

**FIGURE 9:**
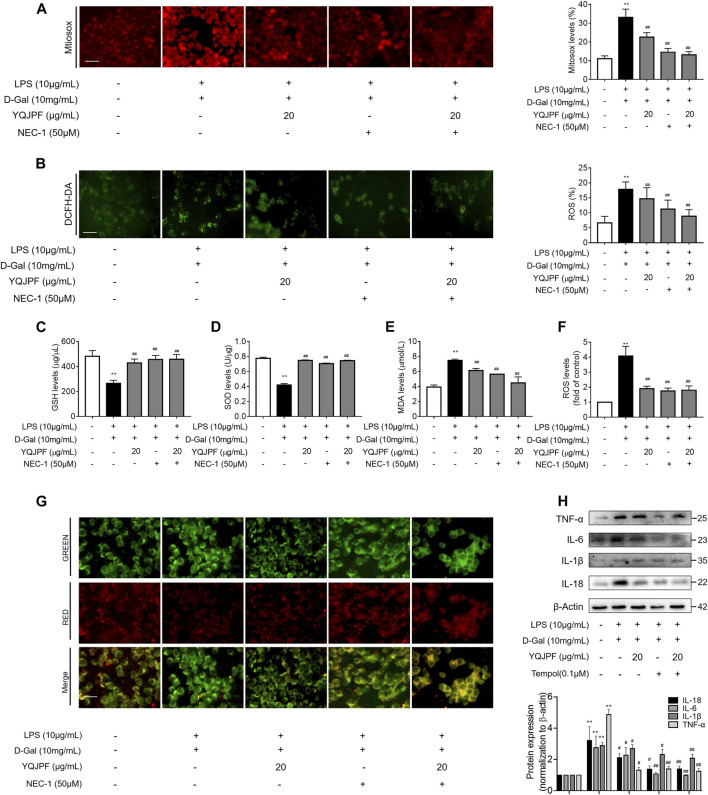
YQJPF inhibited hepatocyte necroptosis through the inhibition of mitochondrial ROS generation and depolarization *in vitro*. Human LO2 cells were treated with LPS + D-Gal for 4 h and further pre-treated with 50 μM Nec-1 for 1 h, followed by YQJPF or atractylone treatment for 24 h. **(A)** Mitochondrial superoxide was detected and quantified through immunofluorescence by using MitoSox Red staining. Scale bar, 50 μm. **(B)** ROS production was assessed and quantified by DCFH-DA staining, Scale bar, 50 μm. **(C–E)** MDA, SOD, and GSH levels were measured by corresponding test kits. **(F)** ROS levels were assessed using ROS test kit. **(G)** Mitochondrial membrane potential was detected by JC-1 staining. Scale bar, 50 μm. **(H)** Western blot and quantitative analysis of the expression of IL-1β, IL-6, TNF-α, and IL-18. Data are expressed as mean ± SD (*n* = 3). *vs. control group, ^#^vs. LPS + D-Gal group; *p < 0.05, **p < 0.01; ^#^ *p* < 0.05, ^##^ *p* < 0.01.

Numerous studies have shown that ROS production is necessary for necroptosis in several cell lines such as macrophages (Koike et al., 2019), fibrosarcoma L929, HeLa, and human embryonic kidney (HEK) 293 T (Luedde et al., 2014; Zhang et al., 2017). To investigate whether YQJPF reduces ROS production in hepatocytes, we first analyzed the effect of YQJPF on intracellular ROS levels. Observation using fluorescence microscope revealed that YQJPF treatment decreases the ROS level in LO2 cells (Figures 9B,C). MitoSox Red staining was used to evaluate the mitochondrial ROS production. As shown in Figure 9A, YQJPF caused a significant decrease in mitochondrial superoxide, which had the same effect as selective RIPK1 inhibitor Nec-1. In addition, we found that pre-treatment of cells with YQJPF or Nec-1 significantly reversed LPS- and D-Gal-induced reduction in intracellular GSH and SOD levels. Additionally, YQJPF or Nec-1 inhibited MDA formation (Figures 9D–F). The effect of YQJPF on MMP was assessed using JC-1 staining, where a high ratio of red/green indicates an increase in MMP. The result indicated that YQJPF or Nec-1 upregulates MMP, recovers LPS- and D-Gal-induced MMP dissipation, and ameliorates mitochondrial depolarization (Figure 9G). The overproduction of ROS triggers serious damages in various cells. We observed that when the cells are pre-treated with ROS scavenger Tempol (10 μM), it could restore necroptosis-associated cell morphology and structure and inhibit the expression of pro-inflammatory cytokines IL-1β, IL-6, TNF-ɑ, and IL-18, as revealed by western blot (Figure 9H), which had the same effect as that of YQJPF. This suggested that YQJPF reduces the level of ROS in hepatocytes and thus plays a critical role in inhibiting cell death. Collectively, these results suggested that YQJPF inhibits necroptosis by reducing the level of mitochondrial ROS and stabilizing MMP in hepatocytes.

### Disruption of Hepatocyte Necroptosis, Instead of Apoptosis, Was Associated with YQJPF Treatment *In Vivo*

**FIGURE 10:**
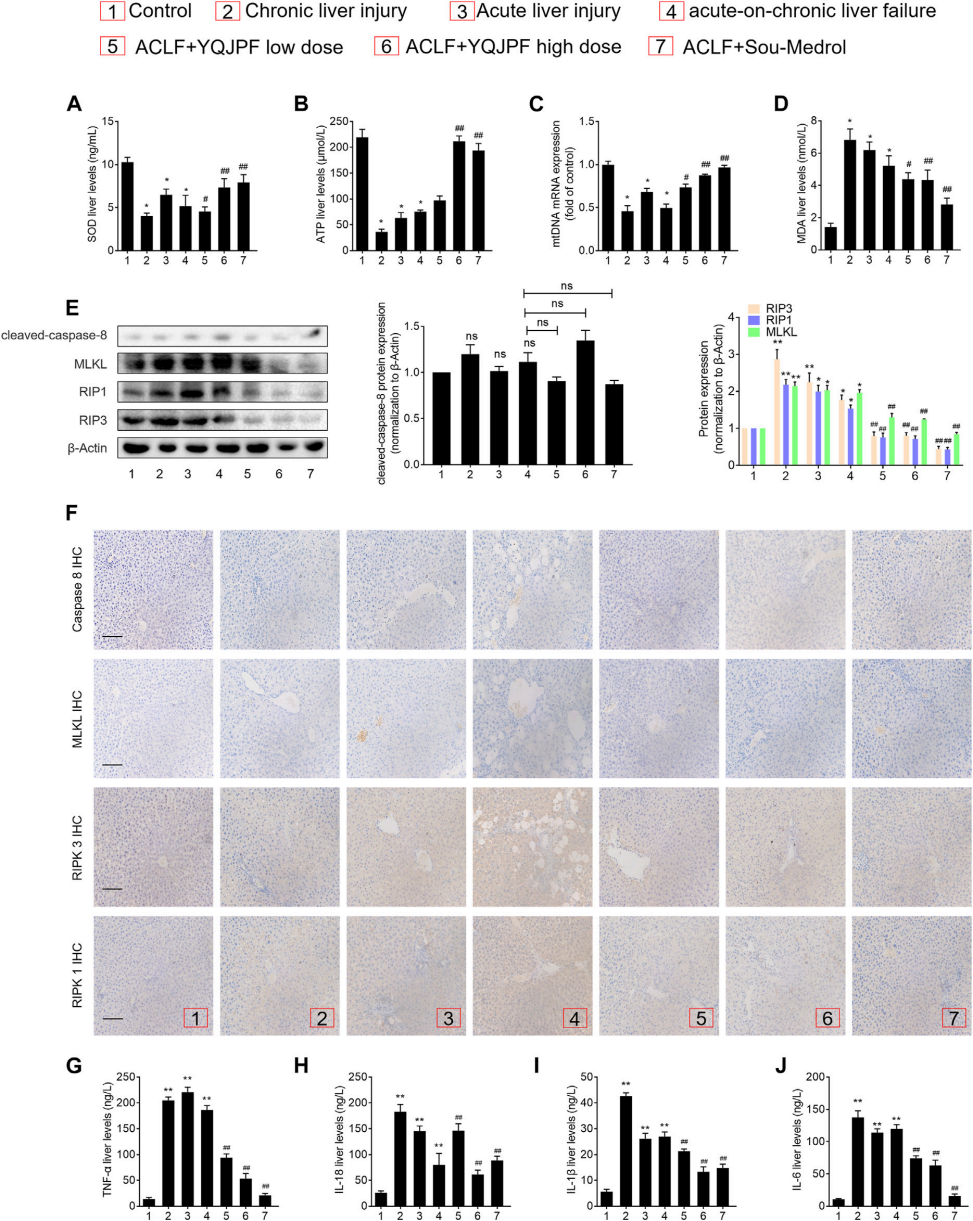
Disruption of hepatocyte necroptosis, and not apoptosis, was required for the action of YQJPF *in vivo.* **(A–D)** MDA, ATP, GSH, and SOD levels in the rat livers were measured. **(E)** Western blot and quantitative analysis of cleaved caspase 8, MLKL, RIPK1, and RIPK3 expression in the rat livers. **(F)** Immunochemical staining and quantitative analysis of caspase 8, MLKL, RIPK1, and RIPK3 in liver tissues. Scale bar, 100 μm. **(G–J)** Expression levels of IL-1β, IL-6, TNF-α, and IL-18 in the rat livers were detected by ELISA. Data are expressed as mean ± SD (*n* = 8) *vs. control group, ^#^vs. ACLF group; *p < 0.05, **p < 0.01; ^#^ *p* < 0.05, ^##^ *p* < 0.01.

The results of *in vivo* studies on the effect of YQJPF on hepatocyte necroptosis were the same as those of *in vitro* results. Protein levels of key caspases did not change under YQJPF treatment. Therefore, reduction in cell death due to YQJPF was independent of apoptosis. Further, we proceeded to investigate whether YQJPF could disrupt hepatocytic death by regulating necroptosis. Western blot analysis of RIPK1, RIPK3, and MLKL, which governed necroptosis, indicated that necroptosis is increased in ACLF *in vivo* (Figure 10E). However, it was weakened by YQJPF treatment. Furthermore, immunochemical staining of RIPK1, RIPK3, and MLKL showed that YQJOF reduces hepatocytic RIPK1, RIPK3, and MLKL levels, which were upregulated in chronic liver injury, acute liver injury, and ACLF model (Figure 10F). Overall, these results demonstrated that YQJPF decreases the levels of RIPK1, RIPK3, and MLKL and decreases ATP production to ameliorate hepatocyte necroptosis instead of apoptosis in ACLF model. Our previous study revealed that JNK1/2-ROS signaling is involved in HSC necroptosis (Jia et al., 2018). To investigate the role of anti-oxidant response in YQJPF-ameliorated hepatocyte necroptosis, we analyzed the effect of YQJPF on SOD and MDA levels in the liver, which are produced during lipid peroxidation. Observation using SOD and MDA detection kits showed that YQJPF treatment increases the anti-oxidant response in the liver (Figure 10A). Interestingly, the levels of ATP and mtDNA decreased significantly in chronic liver injury, acute liver injury, and ACLF model (Figure 10B). Collectively, these results suggest that anti-oxidant response contributes to the effect of YQJPF on hepatocyte necroptosis. Similar to the *in vitro* results, we observed a significant increase in the serum levels of TNF-α, IL-18, IL-1β, and IL-6 in ACLF rats, but the effect was altered in a concentration dependent-manner by YQJPF treatment (Figures 10G–J). These results indicated that YQJPF attenuates hepatic inflammation and liver injury by disrupting hepatocyte necroptosis instead of apoptosis *in vivo*.

## Discussion

The pathobiology of ACLF is characterized by hepatic and systemic inflammation; progressive, unrelenting hepatocyte injury; and hepatocyte death. Hepatocyte death typically follows two patterns: necrosis and apoptosis (Vanlangenakker et al., 2008). In our study, we developed ACLF rat models by administering high dose (1.5 ml/kg body weight) of 1:1 (w/v) mixture of CCl_4_ and olive oil for 3 months followed by 100 μg/kg LPS and 400 mg/kg D-Gal injection. Hepatocyte injury and cell death index were obvious in the *in vivo* model. Through the successful establishment of the disease model, we first confirmed the protective effect of YQJPF on ACLF liver injury. YQJPF could ameliorate CCl_4_-, LPS-, and D-Gal-induced liver injury. Liver tissue morphology improved and serum levels of hepatic ALT, AST, and Tbil decreased after YQJPF treatment. The protective effect of YQJPF was consistent in the *in vitro* experiments. To further clarify the mechanism of action of YQJPF, an *in vitro* hepatocyte injury model was established using LPS (10 μg/ml) and D-Gal (10 mg/ml). YQJPF and atractylone (the main component of YQJPF) could restore the damage to cell viability induced by LPS and D-Gal and decrease ALT, AST, and LDH levels in cell supernatant. These data clearly indicated that YQJPF confers protection against ACLF.

Liver failure is characterized by massive loss of parenchymal cells, and cell death can occur in this pathophysiological context (Schwabe and Luedde, 2018). Necroptosis is defined as the third type of programmed cell death in addition to apoptosis and necrosis (Galluzzi and Kroemer, 2008). It is also known as programmed necrosis or caspase-independent cell death. Necroptosis is believed to trigger a massive inflammatory response that can cause substantial collateral damage to neighboring cells. Findings of a clinical study indicated that circulating RIPK3 is significantly increased in patients with HBV-ACLF and is associated with a clinical outcome (Chen et al., 2020). However, very few studies on drugs inhibiting necroptosis in the liver are available. This study found that YQJPF could attenuate hepatocyte death but has no effect on the inhibition of the caspases three and eight activity. Therefore, YQJPF-induced hepatocytic cell death inhibition did not result from apoptosis. Moreover, a typical cell necroptosis morphology could be identified, and RIPK3 mediates cell death and regulates inflammatory responses (Zhao et al., 2017). Our study showed that the levels of TNF-α, IL-18, IL-1β, and IL-6 were found to be significantly increased in LPS (10 μg/ml)- and D-Gal (10 mg/ml)-induced models. The effect was altered in a concentration-dependent manner after YQJPF treatment. RIPK1, RIPK3, and MLKL were identified as necroptosis regulators (Wang et al., 2012). Further, we assessed expressions of RIPK1 and RIPK3. Western blot analysis, ELISA, and immunochemical staining of RIPK1 and RIPK3 demonstrated that YQJPF decreases hepatocytes necroptosis in the ACLF model. Moreover, YQJPF inhibited the combination expression of RIPK1 and RIPK3 and blocked the formation of necrosome. As expected, YQJPF-induced inhibition of inflammatory cytokines and necrosome formation were enhanced when RIPK1 activity was inhibited by pharmacological inhibitor Nec-1. Obviously, YQJPF inhibited RIPK1/RIPK3-complex-dependent necroptosis in human LO2 cells.

Oxidative stress, a major contributor of liver injury, can be induced by excessive ROS. Studies have shown that ROS may be considerably involved in the pathogenesis of liver diseases (Chen et al., 2016). ROS have a significant effect on necroptosis, particularly the mitochondrial ROS generated in response to TNF/TNFR1 engagement. Conversely, ROS accumulation is also gradually regarded as the executioner and mediator of necroptosis (Zhu et al., 2018). ROS is mainly produced in mitochondria that in turn leads to the damage of organelles or even cell death. In this study, we found significantly decreased levels of intracellular and mitochondrial ROS after YQJPF treatment. Following pre-treatment with the ROS scavenger-Tempol, the YQJPF-induced inhibition of cell inflammatory cytokines was significantly enhanced. Thus, YQJPF could inhibit ROS accumulation. The association between RIPK1 and RIPK3 upregulation and mPTP opening has been illustrated in several studies (He et al., 2011). ROS has been reported as the primary factor that activates mPTP opening and has been implicated in the process of necroptosis. In addition, RIPK1-RIPK3 interaction could upregulate ROS levels and enhance mitochondrial superoxide production. Hence, we identified that the inhibition of RIPK1 with Nec-1 markedly enhances YQJPF-induced alteration of mitochondrial superoxide. Cellular anti-oxidant machinery mainly includes SOD, MDA, and GPx that can effectively scavenge free radicals (Riordan and Williams, 2003). In this study, SOD, GSH, and MDA levels were assessed, and the results suggested that the anti-oxidant response contributes to the effect of YQJPF on hepatocyte necroptosis. Inhibition of RIPK1 by Nec-1 enhanced the effect. Additionally, these findings were confirmed in the rat models.

Traditional Chinese medicine (TCM) pattern is a physiological regulation network, which contains a regulation center, overall effective target, material basis and functional unit. Its mechanism needs to be indicated by means of TCM pattern prevention and treatment characteristics, multiple organ integration effectiveness, and functional unit network so as to guide clinical practice as well as for inspiring basic research. It is urgently to exploring the efficacy mechanism and regulation with the molecular biology and systems biology. In our research, we conducted a deep study of the pathogenesis, TCM pattern feature and efficacy evaluation of TCM in ACLF. And results showed that YQJPF attenuates liver injury in ACLF. Recently, Chen et al.’ reported that flavonoids have been to possess a wide variety of biological activities such as antioxidant activity, anti-inflammatory activity, hepatoprotective effect, antibacterial activity, antiviral activity, anticancer activity, and antidiabetic activity (Chen et al., 2018). Chen et al.’ reported that raspberry treatment could ameliorate H2O2-induced oxidative stress in HepG2 cells *via* Keap1/Nrf2 pathway (Chen et al., 2019). In our study, we found that atractylone (one component of YQJPF) has the similar effect with YQJPF, and inhibited necroptosis of hepatocytes *in vitro*. Which revealed the main component of YQJPF could play such effect.

In summary, YQJPF attenuates liver injury by inhibiting hepatic inflammation and necroptosis of hepatocytes *in vivo* and *in vitro*. Mechanistically, YQJPF inhibits hepatocyte necroptosis by blocking the RIPK1/RIPK3 complex formation followed by relieving mitochondrial dysfunction, which ultimately prevents hepatocytic cell death. Most importantly, we confirmed that YQJPF can be used as a medicine for ACLF treatment and provided a scientific basis for the research on the application of YQJPF in ACLF.

## Data Availability

The raw data supporting the conclusions of this article will be made available by the authors, without undue reservation.

## Abbreviations

ACLF, Acute-on-chronic liver failure; ALP, Alkaline phosphatase; ALT, Alanine aminotransferase; AST, Aspartate aminotransferase; D-Gal, d-galactosamine; ELISA, Enzyme-linked immunosorbent assay, Glb, Globulin, H&E, Hematoxylin and Eosin, HSCs, Hepatic stellate cells, IL-18, Interleukin-18, IL-1β, Interleukin-1β, IL-6, Interleukin-6, LPS, Lipopolysaccharide, MDA, Malondialdehyde, MLKL, Mixed lineage kinase domain-like protein, PBS, Phosphate-buffered saline, RIPK1, Receptor-interacting protein kinases 1, RIPK3, Receptor-interacting protein kinases 3, RT-qPCR, Real-time quantitative polymerase chain reaction, SOD, Superoxide dismutase, TBIL, Total bilirubin, TNF-α, Tumor Necrosis Factor-α, YQJPF, Yiqijianpi Formula, ROS, Reactive oxygen species, Tempol, 4-hydroxy-TEMPO, HBV, Hepatitis B virus, HCV, Hepatitis C virus, Nec-1, Necrotatin, DMEM, Dulbecco’s modified essential medium, FBS, Fetal bovine serum, GAPDH, Glyceraldehyde phosphate dehydrogenase, Co-IP, Co-immunoprecipitation, DCFH-DA, 2′,7′-dichlorodihydrofluorescin diacetate, JC-1, 5.5′,6.6′-tetrachloro-1.1′,3.3′-tetraethylbenzimidazolylcarbocyanine iodide, TEM, Transmission electron microscopy, MMP, Mitochondrial membrane potential, Sou-Medrol, Methylprednisolone

## References

[B1] Asrani S. K. Devarbhavi H. Eaton J. Kamath P. S. (2019). Burden of Liver Diseases in the World. J. Hepatol. 70, 151–171. 10.1016/j.jhep.2018.09.014 30266282

[B2] Bajaj J. S. Moreau R. Kamath P. S. Vargas H. E. Arroyo V. Reddy K. R. (2018). Acute‐on‐Chronic Liver Failure: Getting Ready for Prime Time? Hepatology 68, 1621–1632. 10.1002/hep.30056 29689120

[B3] Chen L. Teng H. Zhang K. Y. Skalicka-Woźniak K. Georgiev M. I. Xiao J. (2016). Agrimonolide and Desmethylagrimonolide Induced HO-1 Expression in HepG2 Cells through Nrf2-Transduction and P38 Inactivation. Front. Pharmacol. 7, 513. 10.3389/fphar.2016.00513 28119605 PMC5223292

[B4] Chen L. Cao Z. Yan L. Ding Y. Shen X. Liu K. (2020). Circulating Receptor-Interacting Protein Kinase 3 Are Increased in HBV Patients with Acute-On-Chronic Liver Failure and Are Associated with Clinical Outcome. Front. Physiol. 11, 526. 10.3389/fphys.2020.00526 32655398 PMC7325886

[B5] Chen L. Li K. Liu Q. Quiles J. L. Filosa R. Kamal M. A. (2019). Protective Effects of Raspberry on the Oxidative Damage in HepG2 Cells through Keap1/Nrf2-dependent Signaling Pathway. Food Chem. Toxicol. 133, 110781. 10.1016/j.fct.2019.110781 31465820

[B6] Chen L. Teng H. Jia Z. Battino M. Miron A. Yu Z. (2018). Intracellular Signaling Pathways of Inflammation Modulated by Dietary Flavonoids: The Most Recent Evidence. Crit. Rev. Food Sci. Nutr. 58, 2908–2924. 10.1080/10408398.2017.1345853 28682647

[B7] Cheng Y. Chen T. Yang X. Xue J. Chen J. (2019). Atractylon Induces Apoptosis and Suppresses Metastasis in Hepatic Cancer Cells and Inhibits Growth In Vivo. Cmar Vol. 11, 5883–5894. 10.2147/cmar.s194795 PMC6607983 31388314

[B8] Engelmann C. Mehta G. Tacke F. (2020). Regeneration in Acute-On-Chronic Liver Failure - the Phantom Lost its Camouflage. J. Hepatol. 72, 610–612. 10.1016/j.jhep.2020.01.003 31953140

[B9] Galluzzi L. Kroemer G. (2008). Necroptosis: a Specialized Pathway of Programmed Necrosis. Cell 135, 1161–1163. 10.1016/j.cell.2008.12.004 19109884

[B10] He S. Liang Y. Shao F. Wang X. (2011). Toll-like Receptors Activate Programmed Necrosis in Macrophages through a Receptor-Interacting Kinase-3-Mediated Pathway. Proc. Natl. Acad. Sci. 108, 20054–20059. 10.1073/pnas.1116302108 22123964 PMC3250173

[B11] Jia L. Xue R. Zhu Y. Zhao J. Li J. He W. P. (2020). The Efficacy and Safety of Methylprednisolone in Hepatitis B Virus-Related Acute-On-Chronic Liver Failure: a Prospective Multi-Center Clinical Trial. BMC Med. 18, 383. 10.1186/s12916-020-01814-4 33287816 PMC7722342

[B12] Jia Y. Wang F. Guo Q. Li M. Wang L. Zhang Z. (2018). Curcumol Induces RIPK1/RIPK3 Complex-dependent Necroptosis via JNK1/2-ROS Signaling in Hepatic Stellate Cells. Redox Biol. 19, 375–387. 10.1016/j.redox.2018.09.007 30237126 PMC6142373

[B13] Jin H. Lian N. Zhang F. Chen L. Chen Q. Lu C. (2016). Activation of PPARgamma/P53 Signaling Is Required for Curcumin to Induce Hepatic Stellate Cell Senescence. Cell Death Dis 7, e2189. 10.1038/cddis.2016.92 27077805 PMC4855671

[B14] Koike A. Hanatani M. Fujimori K. (2019). Pan-caspase Inhibitors Induce Necroptosis via ROS-Mediated Activation of Mixed Lineage Kinase Domain-like Protein and P38 in Classically Activated Macrophages. Exp. Cel Res. 380, 171–179. 10.1016/j.yexcr.2019.04.027 31039349

[B15] Lian Y. Xu L. Tan S. Xiao Q. Sun W. Shen J. (2015). Effects of Treatment on T Lymphocyte Frequency with Principle of Strengthening Vital Qi and the Spleen Based on the Mechanism of Deficiency in Vital Qi in Patients. *JETCM* Chin. 24, 2106–2108.

[B16] Lin J. Kumari S. Kim C. Van T.-M. Wachsmuth L. Polykratis A. (2016). RIPK1 Counteracts ZBP1-Mediated Necroptosis to Inhibit Inflammation. Nature 540, 124–128. 10.1038/nature20558 27819681 PMC5755685

[B17] Luedde T. Kaplowitz N. Schwabe R. F. (2014). Cell Death and Cell Death Responses in Liver Disease: Mechanisms and Clinical Relevance. Gastroenterology 147, 765–783. 10.1053/j.gastro.2014.07.018 25046161 PMC4531834

[B18] Newton K. Dugger D. L. Maltzman A. Greve J. M. Hedehus M. Martin-Mcnulty B. (2016). RIPK3 Deficiency or Catalytically Inactive RIPK1 Provides Greater Benefit Than MLKL Deficiency in Mouse Models of Inflammation and Tissue Injury. Cell Death Differ 23, 1565–1576. 10.1038/cdd.2016.46 27177019 PMC5072432

[B19] Riordan S. M. Williams R. (2003). Mechanisms of Hepatocyte Injury, Multiorgan Failure, and Prognostic Criteria in Acute Liver Failure. Semin. Liver Dis. 23, 203–215. 10.1055/s-2003-42639 14523674

[B20] Sarin S. K. Choudhury A. (2016). Acute-on-chronic Liver Failure: Terminology, Mechanisms and Management. Nat. Rev. Gastroenterol. Hepatol. 13, 131–149. 10.1038/nrgastro.2015.219 26837712

[B21] Schwabe R. F. Luedde T. (2018). Apoptosis and Necroptosis in the Liver: a Matter of Life and Death. Nat. Rev. Gastroenterol. Hepatol. 15, 738–752. 10.1038/s41575-018-0065-y 30250076 PMC6490680

[B22] Vanlangenakker N. Berghe T. Krysko D. Festjens N. Vandenabeele P. (2008). Molecular Mechanisms and Pathophysiology of Necrotic Cell Death. Cmm 8, 207–220. 10.2174/156652408784221306 18473820

[B23] Wang F. S. Fan J. G. Zhang Z. Gao B. Wang H. Y. (2014). The Global Burden of Liver Disease: the Major Impact of China. Hepatology 60, 2099–2108. 10.1002/hep.27406 25164003 PMC4867229

[B24] Wang Z. Jiang H. Chen S. Du F. Wang X. (2012). The Mitochondrial Phosphatase PGAM5 Functions at the Convergence Point of Multiple Necrotic Death Pathways. Cell 148, 228–243. 10.1016/j.cell.2011.11.030 22265414

[B25] Weinlich R. Oberst A. Beere H. M. Green D. R. (2017). Necroptosis in Development, Inflammation and Disease. Nat. Rev. Mol. Cel Biol 18, 127–136. 10.1038/nrm.2016.149 27999438

[B26] Xiang X. Feng D. Hwang S. Ren T. Wang X. Trojnar E. (2020). Interleukin-22 Ameliorates Acute-On-Chronic Liver Failure by Reprogramming Impaired Regeneration Pathways in Mice. J. Hepatol. 72, 736–745. 10.1016/j.jhep.2019.11.013 31786256 PMC7085428

[B27] Xie F. Dong J. Zhu Y. Wang K. Liu X. Chen D. (2019). HIF1a Inhibitor Rescues Acute-On-Chronic Liver Failure. Ann. Hepatol. 18, 757–764. 10.1016/j.aohep.2019.03.007 31402229

[B28] Yao H. Huang X. Xie Y. Huang X. Ruan Y. Lin X. (2018). Identification of Pharmacokinetic Markers for Guanxin Danshen Drop Pills in Rats by Combination of Pharmacokinetics, Systems Pharmacology, and Pharmacodynamic Assays. Front. Pharmacol. 9, 1493. 10.3389/fphar.2018.01493 30622470 PMC6308302

[B29] Zhang F. Zhang Z. Kong D. Zhang X. Chen L. Zhu X. (2014a). Tetramethylpyrazine Reduces Glucose and Insulin-Induced Activation of Hepatic Stellate Cells by Inhibiting Insulin Receptor-Mediated PI3K/AKT and ERK Pathways. Mol. Cell Endocrinol. 382, 197–204. 10.1016/j.mce.2013.09.020 24071517

[B30] Zhang Y. Su S. S. Zhao S. Yang Z. Zhong C. Q. Chen X. (2017). RIP1 Autophosphorylation Is Promoted by Mitochondrial ROS and Is Essential for RIP3 Recruitment into Necrosome. Nat. Commun. 8, 14329. 10.1038/ncomms14329 28176780 PMC5309790

[B31] Zhang Z. Zhang F. Wang Y. Du Y. Zhang H. Kong D. (2014b). Traditional Chinese Medicine for Stable Angina Pectoris via TCM Pattern Differentiation and TCM Mechanism: Study Protocol of a Randomized Controlled Trial. Trials 15, 422. 10.1186/1745-6215-15-422 25359307 PMC4233055

[B32] Zhao J. Zhang J.-Y. Yu H.-W. He Y.-L. Zhao J.-J. Li J. (2012). Improved Survival Ratios Correlate with Myeloid Dendritic Cell Restoration in Acute-On-Chronic Liver Failure Patients Receiving Methylprednisolone Therapy. Cell Mol Immunol 9, 417–422. 10.1038/cmi.2011.51 22231552 PMC4002325

[B33] Zhao Q. Yu X. Zhang H. Liu Y. Zhang X. Wu X. (2017). RIPK3 Mediates Necroptosis during Embryonic Development and Postnatal Inflammation in Fadd -Deficient Mice. Cel Rep. 19, 798–808. 10.1016/j.celrep.2017.04.011 28445730

[B34] Zhu P. Hu S. Jin Q. Li D. Tian F. Toan S. (2018). Ripk3 Promotes ER Stress-Induced Necroptosis in Cardiac IR Injury: A Mechanism Involving Calcium overload/XO/ROS/mPTP Pathway. Redox Biol. 16, 157–168. 10.1016/j.redox.2018.02.019 29502045 PMC5952878

